# Interleukin 6 Receptor Is an Independent Prognostic Factor and a Potential Therapeutic Target of Ovarian Cancer

**DOI:** 10.1371/journal.pone.0118080

**Published:** 2015-02-06

**Authors:** Aki Isobe, Kenjiro Sawada, Yasuto Kinose, Chifumi Ohyagi-Hara, Erika Nakatsuka, Hiroshi Makino, Tomonori Ogura, Tomoko Mizuno, Noriko Suzuki, Eiichi Morii, Koji Nakamura, Ikuko Sawada, Aska Toda, Kae Hashimoto, Seiji Mabuchi, Tsuyoshi Ohta, Ken-ichirou Morishige, Hirohisa Kurachi, Tadashi Kimura

**Affiliations:** 1 Department of Obstetrics and Gynecology, Osaka University Graduate School of Medicine, Suita, Osaka, Japan; 2 Department of Obstetrics and Gynecology, JCHO Osaka Hospital, Osaka, Japan; 3 Department of Obstetrics and Gynecology, Gifu University Graduate School of Medicine, Gifu, Japan; 4 Department of Pathology, Osaka University Graduate School of Medicine, Suita, Osaka, Japan; 5 Department of Obstetrics and Gynecology, Yamagata University Faculty of Medicine, Yamagata, Japan; Garvan Institute of Medical Research, AUSTRALIA

## Abstract

Ovarian cancer remains the most lethal gynecologic cancer and new targeted molecular therapies against this miserable disease continue to be challenging. In this study, we analyzed the expressional patterns of Interleukin-6 (IL-6) and its receptor (IL-6R) expression in ovarian cancer tissues, evaluated the impact of these expressions on clinical outcomes of patients, and found that a high-level of IL-6R expression but not IL-6 expression in cancer cells is an independent prognostic factor. In *in vitro* analyses using ovarian cell lines, while six (RMUG-S, RMG-1, OVISE, A2780, SKOV3ip1 and OVCAR-3) of seven overexpressed IL-6R compared with a primary normal ovarian surface epithelium, only two (RMG-1, OVISE) of seven cell lines overexpressed IL-6, suggesting that IL-6/IL-6R signaling exerts in a paracrine manner in certain types of ovarian cancer cells. Ovarian cancer ascites were collected from patients, and we found that primary CD11b^+^CD14^+^ cells, which were predominantly M2-polarized macrophages, are the major source of IL-6 production in an ovarian cancer microenvironment. When CD11b^+^CD14^+^ cells were co-cultured with cancer cells, both the invasion and the proliferation of cancer cells were robustly promoted and these promotions were almost completely inhibited by pretreatment with anti-IL-6R antibody (tocilizumab). The data presented herein suggest a rationale for anti-IL-6/IL-6R therapy to suppress the peritoneal spread of ovarian cancer, and represent evidence of the therapeutic potential of anti-IL-6R therapy for ovarian cancer treatment.

## Introduction

Ovarian cancer is the leading cause of death from gynecologic malignancies. Recent convincing data support the involvement of the inflammatory stromal microenvironment, caused by over-expression of cytokines or chemokines, in promoting ovarian tumorigenesis, cancer progression and resistance to chemotherapies.[1] Therefore, targeting these cytokines from the stromal microenvironment may offer a promising therapeutic strategy to improve the management of patients with ovarian cancer.

Among the cytokines reported so far, Interleukin-6 (IL-6) is one of the pivotal immunoregulatory cytokines present in the ovarian cancer microenvironment; it induces several pathways leading to tumor proliferation, angiogenesis and chemoresistance.[2] Higher serum and ascites levels of IL-6 have been found in patients with ovarian cancer than in patients with other malignancies, and levels have been shown to correlate with the extent of disease and poor clinical outcome.[3–5] Although Rath et al. recently showed that IL6-R expression is highly expressed in ovarian cancer tissues compared with normal tissues or benign diseases,[6] the clinical impact of IL6-R expression in ovarian cancer species has not been examined. Therefore, we were encouraged to investigate the clinical values of IL-6 and IL-6R in ovarian cancer tissues using the tissue microarrays (TMAs) we constructed and the corresponding clinical data.

It appears that antagonizing IL-6/IL-6R signaling may have therapeutic activity in patients with ovarian cancer through the inhibition of a tumor-promoting cytokine network. Indeed, targeted anti-IL-6 antibody therapy has been used in clinical trials and found to be well tolerated in patients of several cancers, including ovarian cancer.[7] Tocilizumab (Chugai Pharmaceutical, Shizuoka, Japan), is a humanized anti-human IL-6R antibody and binds to the IL-6-binding site of human IL-6R. It is known to competitively inhibit IL-6/IL-6R signaling and completely neutralizes IL-6 activities.[8, 9] A series of clinical studies has successfully shown that the suppression of IL-6/IL-6R signaling by tocilizumab is therapeutically effective in alleviate Castleman’s disease and rheumatoid arthritis.[10, 11] Given its success in treating these diseases, tocilizumab may prove useful in treating IL-6–related cancers and we were motivated to elucidate the therapeutic potential of tocilizumab against ovarian cancer.

Although not only ovarian cancer cells but tumor-associated macrophages have been reported to produce IL-6,[12, 13] it remains debatable whether increased IL-6 levels in patients with ovarian cancer are produced by the tumor itself or mainly by host tissues. The majority of patients with ovarian cancer at advanced stages present peritoneal metastatic diseases, often accompanied by massive ascites.[14] Massive ascites of patients consist of not only cancer cells but also fibroblasts, endothelial cells and predominantly immune cells, all of which are crucial for cancer growth, progression and metastasis.[15] Peritoneal macrophages are thought to play a pivotal role in this context, as is evidenced by several studies finding that macrophage depletion in peritoneal ovarian cancer models suppresses cancer progression and accumulation of ascites.[16, 17] Macrophages that infiltrate tumor tissues, which are referred to as tumor-associated macrophages (TAM), are well-known contributors to tumor progression and are associated with the poor prognosis of various cancers.[18, 19] Since TAMs are known to release various proangiogenic cytokines and growth factors, we hypothesized that macrophages could be one of potential responsible sources of enriched IL-6 accumulation in ovarian cancer ascites.

Against this background, we attempted to analyze the expressional pattern of IL-6R as well as using ovarian cancer TMAs and to evaluate the impact of these expressions on the clinical outcomes of patients. Ovarian cancer ascites were collected from patients who underwent surgery and we found that primary CD11b^+^CD14^+^ cells, which were predominantly M2-polarized TAMs, were the major source of IL-6 production in an ovarian tumor microenvironment and robustly promoted ovarian cancer invasion and proliferation. The data presented herein suggest a rationale for anti-IL-6/IL-6R therapy to suppress the peritoneal spread of ovarian cancer and provide evidence of the therapeutic potential of anti-IL-6R therapy to cure this miserable disease.

## Materials and Methods

### Ethics Statement

Patient samples were obtained with written informed consent in accordance with ethics committee requirements from the institutes and the Declaration of Helsinki. Institutional Review Board (IRB) of Osaka University Graduate School of Medicine approved this study protocol on 2011/02/15 (No. 10064). IRB of Gifu University Graduate School of Medicine approved it on 2012/7/2 (No. 26–50).

### Materials

Antibodies against IL-6 (R-49L), IL-6R (C-20) and total STAT3 (C-20) were from Santa Cruz Biotechnology (Santa Cruz, CA). Recombinant human IL-6 was obtained from Chemicon (Temecula, CA) and recombinant human sIL-6R was from R&D systems (Minneapolis, MN). Antibodies against total-p44/42 MAPK (ERK 1/2) (3A7), phosphorylated (Thr202/Tyr204) p44/42 MAPK (p-ERK 1/2) (E10), phosphorylated (Tyr705) STAT3 (p-STAT3) and β-actin were from Cell Signaling (Beverly, MA). Humanized anti-human IL-6 receptor antibody, tocilizumab, was kindly provided by Chugai Pharmaceutical Co. Ltd. (Shizuoka, Japan) and non-immune human IgG was purchased from Jackson Immuno Research (West Grove, PA). Antibody against CD68 (25747–1-AP) was purchased from Proteintech group Inc. (Chicago, IL, USA). Growth factor-reduced basement membrane proteins (Matrigel) and 24-transwell chambers were purchased from BD Biosciences (Bedford, MA).

### Cell culture

The ovarian cancer cell lines, A2780 and CaOV3, were purchased from American Type Culture Collection (Rockville, MD). RMUG-S, OVISE and RMG-1 cell lines were obtained from the Health Science Research Resources Bank (Osaka, Japan). OVCAR-3 cells were from Cell Resource Center for Biomedical Research, Institute of Development, Aging and Cancer, Tohoku University (Sendai, Japan). SKOV3ip1 cells were kindly provided by Dr. Ernst Lengyel (University of Chicago, Chicago, IL). Cells were cultured in DMEM supplemented with 10% fetal bovine serum and 1000 U/ml penicillin/streptomycin, incubated in 95% air / 5% CO_2_ at 37°C. A2780, CaOV3, OVCAR-3 and SKOV3ip1 cells were established from serous papillary adenocarcinomas of human ovary. RMUG-S cells were from a human ovarian mucinous cystadenocarcinoma. OVISE and RMG-1 cells were from clear cell carcinomas of human ovary. Primary ovarian surface epithelium (OSE) cells were obtained from normal ovarian specimens of patients who underwent surgery for benign conditions at Osaka University Hospital. Written informed consent was obtained from each patient before their operation. The culture method followed the protocol written by Shepherd TG, et al.[20] Subcultures were obtained by trypsinization and were used for experiments at passages 3 to 5.

### Patients and tissue microarray

Ovarian carcinoma samples were collected from patients treated at Gifu University Hospital (Gifu, Japan) between 2006 and 2011 and were used to construct the tissue microarray (TMA) slides. Briefly, resected specimens were fixed in 10% buffered formalin and representative regions were processed for paraffin embedding. From the corresponding regions in paraffin blocks, tissue cores (diameter, 3 mm) were removed using a hollow needle, arrayed in paraffin blocks (TMA) and sliced (4 μm thick) onto slides. Institutional Review Board approval was obtained from the Institute. Satisfactory tissue cores were finally obtained from 94 patients, and the corresponding clinical data were collected.

### Immunohistochemistry

The TMA slides were deparaffinized in xylene and dehydrated with 100% ethanol before antigen unmasking was performed by boiling the slides in Target Retrieval Solution (pH9.0) (Dako, Glostrup, Denmark). After being placed in 3% H_2_O_2_ and being blocked with blocking solution (Dako), they were incubated with the primary IL-6 and IL-6R antibody at 1:200 for 18 hours at 4°C and with the primary CD68 antibody at 1:200 for 30 min at room temperature. After washing with PBS, they were stained using the Envision system (Dako) and then counterstained with Mayer’s hematoxylin. Placentae complicated with chorioamnionitis were used as a positive control. Slides were intensively examined by two independent qualified pathologists (EM, YK) without knowledge of clinical outcomes and each sample was scored based on the percentage of positive cells (0, <25%; 1, ≥25%) and the intensity of the staining (0, none; 1, weak; 2, strong). “High” expression of IL-6 was defined if the composition score of density and intensity was ≥1. “Low” expression was defined if the composition score of density and intensity scores was 0. “High” expression of IL-6R was defined if the composition score of density and intensity was = 2. “Low” expression was defined if the composition score of density and intensity scores was ≤1.

### Real Time Reverse Transcription (RT)-PCR analysis

Total RNA was isolated with Trizol reagent (Invitrogen, Carlsbad, CA). One μg of total RNA was reverse-transcribed with ReverTra Ace qPCR RT Master Mix (Toyobo, Japan). For quantitative mRNA expression analysis, TaqMan RT-PCR was performed on the StepOnePlus™Real-Time PCR system following the manufacturer's instructions. TaqMan Probe-Based Gene Expression Assays were used for IL-6; Hs 00174131_m1 and IL-6R; Hs 01075667_m1 (Applied Biosystems). GAPDH was used as an internal control. Relative levels of mRNA gene expression were calculated using the 2^-*ΔΔCT*^ method as described previously.[21]

### RT-PCR analysis

cDNA was synthesized from 1 μg RNA using a ReverTra Ace qPCR RT Master Mix (Toyobo, Osaka, Japan) with random primers. For IL-6R expression, the primers were designed to anneal to the region splicing out from IL-6R in order to detect not only a membrane-bound form (IL-6R) but also a soluble form (sIL-6R). The primer sequences and the expected PCR products were as follows; sense IL-6R, 5′-TCCACCCCCATGCAGGCACT-3′ (bases 1419 to 1438) and anti-sense IL-6R; 5′-GTGCCACCCAGCCAGCTATC-3′ (bases 1840 to 1859), size; IL-6R, 441 bp, sIL-6R, 341 bp. β-actin-sense: 5’-CGTGACATTAAGGAGCTGTG-3’, β-actin-anti-sense: 5’-GCTCAGGAGGAGCAATGATCTTGA-3’, size 376 bp. The sequences of cDNA were referred to GenBank (accession no. X12830 for IL-6R and sIL-6R and NM_001101 for β-actin). The cDNA templates were PCR-amplified with Taq PCR master mix (Qiagen, Valencia, CA) containing 1 mM each of dATP, dCTP, dGTP and dTTP, and 2.5 U Taq DNA polymerase, and each specific primer at 0.2 μM under the following conditions: 35 cycles of 94°C for 45s, 60°C for 45s and 72°C for 60s. The products were electrophoresed on 2% agarose gels and visualized by ethidium bromide staining and ultraviolet illumination.

### Western Blot Analysis

A total of 4 × 10^5^ cells were plated onto 6-well plates and lysed with 1 × Cell Lysis Buffer (Cell Signaling). Lysates (30 μg) were separated by 5–20% SDS-PAGE and transferred to polyvinylidene difluoride membranes, followed by incubation with the primary antibodies (p-STAT3, 1:1000 in 5% bovine serum albumin (BSA); STAT3, 1:1000 in 5% BSA; p-ERK, 1:2000 in 5% BSA, ERK, 1:2000 in 5% BSA; β-Actin, 1:10000 in 5% BSA) and then with a corresponding secondary horseradish peroxidase conjugated IgG. The proteins were visualized with Western Lightning Plus ECL (PerkinElmer Life Science, Waltham, MA).

### Cell Proliferation Assay

SKOV3ip1 or RMG1 cells were plated in 24-well plates (1 x 10^4^ cells / well) in 10% FBS/DMEM for 24 h and then incubated in 0.1% BSA/DMEM in the presence or absence of IL-6 with or without anti-IL-6R antibody for 72 h. Cell proliferation was evaluated by a modified MTS assay using a Cell Titer 96AQ kit (Promega, Madison, WI), in accordance with the manufacturer’s instructions. In co-culture experiments, polycarbonate filters with 1-μm pores (cells cannot pass through the filters) were placed onto 24-well plates and equal numbers of primary cells were seeded as a stimulant. Cell proliferation was expressed as the ratio of the number of viable cells.

### Matrigel invasion assay

Briefly, polycarbonate filters with 8-μm pores were coated with 25 μg matrigel (BD Biosciences). SKOV3ip1 or RMG1 cells (1 x 10^5^ cells / well) were seeded onto the upper chambers in 0.1% BSA/DMEM and incubated in the same medium containing IL-6 as a chemoattractant in the bottom chamber for 72 h.Thereafter, filters were fixed and stained with Carazzi’s Hematoxylin solution. Noninvading cells were removed using a cotton swab, and invading cells on the underside of the filter were counted using an inverted microscope. Five orbital microscopic fields from each filter were randomly chosen. In co-culture experiments, equal numbers of primary cells were seeded in the bottom chamber as a chemoattractant.

### Enzyme linked immunosorbant assay (ELISA)

For the quantitative assay of human VEGF-A, SKOV3ip1 cells (1 x 10^5^ cells) were cultured under serum-free medium in the presence or absence of IL-6 for 72 h. Anti-IL-6R antibody (10 μg/ml) or an equivalent amount of control IgG was concurrently applied. Thereafter, conditioned culture media were collected and stored at -80°C until analysis. Human VEGF-A Platinum ELISA kit (eBiosecience, SanDiego, CA) was used to determine the concentration of VEGF-A, in accordance with the manufacturer’s protocol, with a sensitivity of 7.9 pg/mL. For the quantitative assay of human IL-6 or human sIL-6R, 1 x 10^5^ of SKOV3ip1 cells and primary cells were cultured under serum-free medium for 72 h and these concentrations in culture medium was measured using Human IL-6 ELISA Ready-SET-GO with a sensitivity of 2 pg/mL or Human sIL-6R Instant ELISA (eBioscience) with a sensitivity of 10 pg/mL.

### Isolation of primary cells from ovarian cancer ascites

Patient samples were obtained with written informed consent in accordance with Osaka University Hospital ethics committee requirements and the Declaration of Helsinki. Ascites were collected aseptically, and cells were isolated by standard Ficoll-Paque density-gradient (Amersham Biosciences, Uppsala, Sweden). Thereafter, CD11b positive (CD11b^+^) cells as well as CD14 positive (CD14^+^) cells were purified by positive selection using magnetic-activated cell sorting (MACS) technology (Miltenyi Biotec, Bergisch Gladbach, Germany). Finally, CD11b^-^ cells, CD11b^+^CD14^-^ cells and CD11b^+^CD14^+^ cells were collected. Cells were cultured in 20% FBS RPMI 1640 medium and used for experiments at passages 2 to 3.

### Fluorescence-activated cell sorting (FACS) analysis

For cell surface staining, single-cell suspensions were incubated with phycoerythrin (PE)-conjugated anti-CD11b monoclonal antibody (ICRF44) (BD Biosciences), allophycocyanin (APC)-conjugated anti-CD14 monoclonal antibody (M5E2) (BD Biosciences), Alexa Fluor-488 conjugated anti-CD68 monoclonal antibody (y1/82A) (Miltenyi Biotec) and eFluor-450 conjugated anti-CD206 antibody (19.2) (eBioscience, San Die go, CA) for 30 minutes at 4°C. After washing, cells were resuspended in 500 μL of 1% BSA/PBS. The stained cells were run on a FACSCanto II flow cytometer (BD Biosciences). Data were analyzed using the FlowJo software program (TreeStar, Ashland, OR).

### Statistical analysis

Statcel version 3 (OMS-Publishing Inc., Saitama, Japan) and JMP version 10.0.2 (SAS Institute Japan Ltd., Tokyo, Japan) were used for statistical analyses. Differences were analyzed using the Mann-Whitney U test. Survival estimates were computed using the Kaplan-Meier method, and comparisons between groups were analyzed using the log-rank test. Multivariable analysis was performed using a Cox proportional-hazards regression model. Differences were considered statistically significant at the two-tailed *P* < 0.05 level.

## Results

### High expression of IL-6 receptor is an independent prognostic marker of ovarian cancer patients

**Fig 1:**
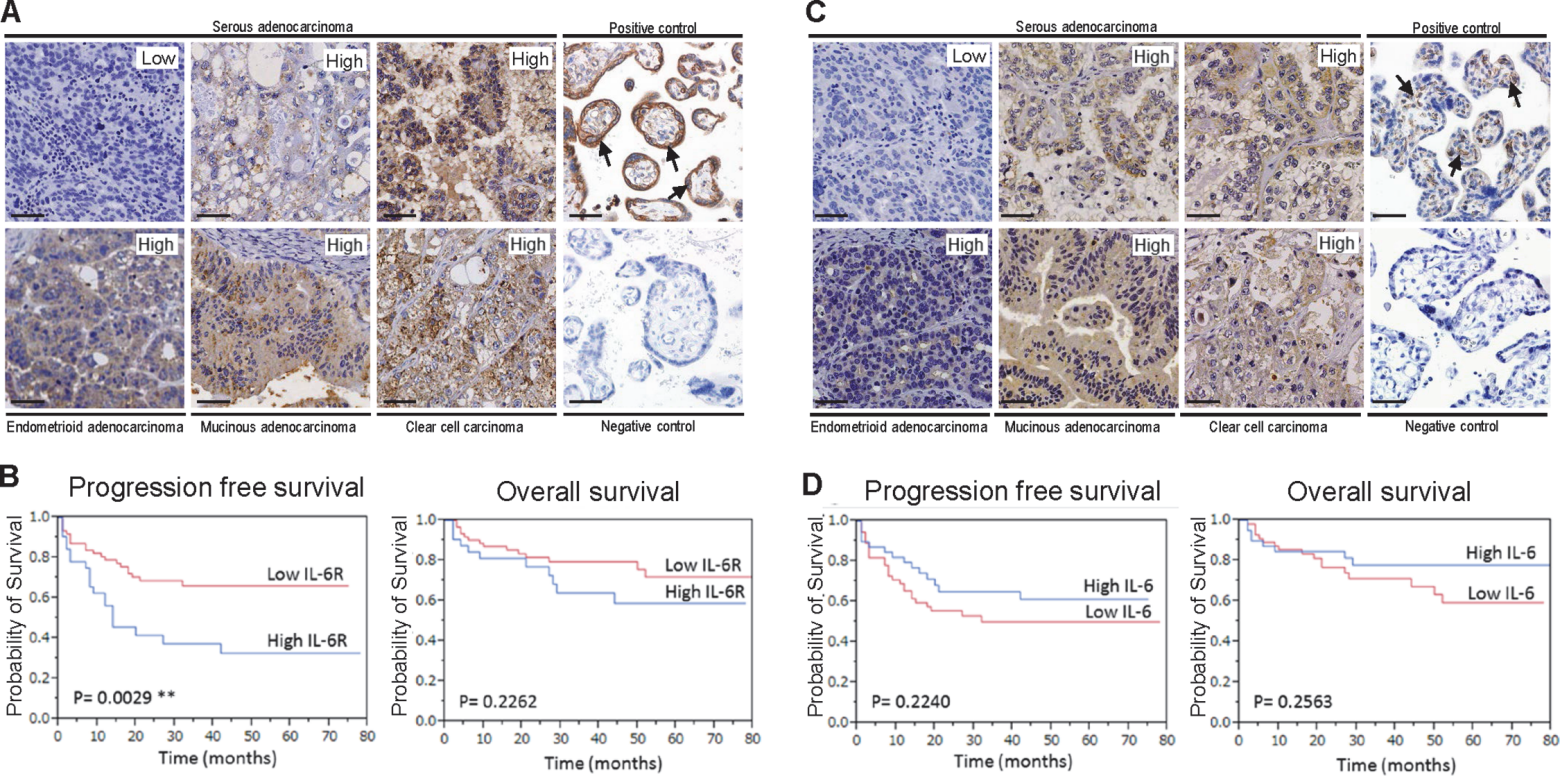
IL-6 receptor expression but not IL-6 expression correlates with poor prognosis in patients with ovarian cancer. (A) Immunohistochemical staining of tissue microarrays with malignant ovarian tissue sections. Representative areas of four different ovarian cancers stained using an anti-human IL-6R antibody and scored as “Low” or “High”. Placentae complicated with chorioamnionitis were used as a positive control. Arrows indicate membranous staining in trophoblasts. A negative control is nonimmune sera. (B) IL-6 receptor expression correlates with poor prognosis in patients with ovarian cancer. Kaplan-Meier curves of progression free survival (*left*) and overall survival (*right*) of ovarian cancer patients treated at Gifu University Hospital (n = 94). (C) Representative areas of four different ovarian cancers stained using an anti-human IL-6 antibody and scored as “Low” or “High”. Placentae complicated with chorioamnionitis were used as a positive control. Arrows indicate cytoplasmic staining in villous mesenchymal cells. A negative control is nonimmune sera. (D) IL-6 expression did not affect the prognosis in patients with ovarian cancer. Kaplan-Meier curves of progression free survival (*left*) and overall survival (*right*) of patients. Original magnification, ×200. Scale bar in each panel represents 50 μm.

In order to assess the therapeutic potential of IL-6R, we established TMA slides from 94 Japanese ovarian cancer patient. The characteristics of patients are summarized in **Table 1**. IL-6R expression was evaluated by immunohistochemistry and each sample was scored based on the percentage of positive cells and the intensity of the staining. Typically, clear membranous staining was seen in the cases of positive IL-6R expression (**Fig. 1A**). Of 94 patients, 32 (34.0%) showed “high” IL-6R expression, including 14 of 34 serous (41.1%), 3 of 16 mucinous (18.8%), 1 of 11 endometrioid (9.1%), 10 of 20 clear cell (50.0%) and 4 of 13 other (30.8%) ovarian carcinomas. In contrast, 62 (66.0%) cases expressed “low” or negative IL-6R expression. Among patients with ovarian cancer, those who had “high” IL-6R expression showed significantly worse PFS than those who had “low” or negative IL-6R expression (PFS, 23.8 vs. 32.3 months, *P* = 0.0029, **Fig. 1B**). Similarly, IL-6 expression was evaluated by immunohistochemistry and each sample was scored. Cytoplasmic staining was seen in the cases of positive IL-6 expression (**Fig. 1C**). Of 94 patients, 39 (41.4%) showed “high” IL-6 expression, including 13 of 34 serous (38.2%), 8 of 16 mucinous (50.0%), 5 of 11 endometrioid (45.5%), 11 of 20 clear cell (55.0%) and 2 of 13 other (15.4%) ovarian carcinomas. In contrast, 55 (58.5%) cases expressed “low” or negative IL-6 expression. Although the prognostic value of IL-6 was examined by Kaplan-Meier method and comparisons performed using the log-rank test, IL-6 expression failed to show any significant correlations with prognoses of patients (**Fig. 1D**). For a multivariate analysis was performed to select a model for survival with multiple predictors. Age, FIGO stage, histological type, residual tumor at the time of surgery, “high” IL-6 expression and “high” IL-6R expression were entered in this model. The final model included “high” IL-6R expression but not IL-6 expression is a significant independent predictor for reduced PFS in ovarian cancer patients (*P* = 0.017, *HR*; 2.388 (1.171–4.895), **Table 2**) along with clinical stage and surgeries.

**Table 1:** Characteristics of the patients (n = 94).

Median age, y (range)	56 (20–88)
Median observation time of patients alive, Mo (range)	29 (2–145)
FIGO stage, n (%)	
I	40 (42.6)
II	5 (5.3)
III	33 (35.1)
IV	16 (17.0)
Histologic subtype, n (%)	
Serous adenocarcinoma	34 (36.2)
Clear cell carcinoma	20 (21.3)
Endometrial adenocarcinoma	11 (11.7)
Mucinous adenocarcinoma	16 (17.0)
Unclassified adenocarcinoma	4 (4.3)
Mixed epithelial tumors	3 (3.2)
Carcinosarcoma	2 (2.1)
Immature teratoma	2 (2.1)
Undifferentiated carcinoma	1 (1.1)
Others	1 (1.1)
Residual tumor (cm), n (%)	
≦1	60 (63.8)
>1	32 (34.0)
unknown	2 (2.1)
Chemotherapy, n (%)	
Taxane/platinum	61 (64.9)
Others	9 (9.6)
None	24 (25.5)

**Table 2:** Multivariate analysis for progression free survival of the patients.

	n (%)	HR	95% CI	*P* value
Age, yrs				
<70	75 (79.8)	1		
≥70	19 (20.2)	0.531	0.228–1.133	0.104
FIGO Stage				
I-II	45 (47.9)	1		
III-IV	49 (52.1)	12.92	4.080–57.080	<0.001
Histology				
Non-serous	60 (63.8)	1		
Serous	34 (36.2)	0.926	0.461–1.893	0.831
Primary surgical outcome				
Optimal	60 (63.8)	1		
Suboptimal	34 (36.2)	2.262	1.301–5.513	0.007
IL-6 stain				
Low	62 (66.0)	1		
High	32 (34.0)	0.635	0.303–1.283	0.209
IL-6R stain				
Low	72 (76.6)	1		
High	22 (23.4)	2.388	1.171–4.895	0.017

HR, hazard ratio;

95% CI, 95% confidence interval.

### IL-6 receptor but not IL-6 is highly expressed in ovarian cancer cell lines

**Fig 2:**
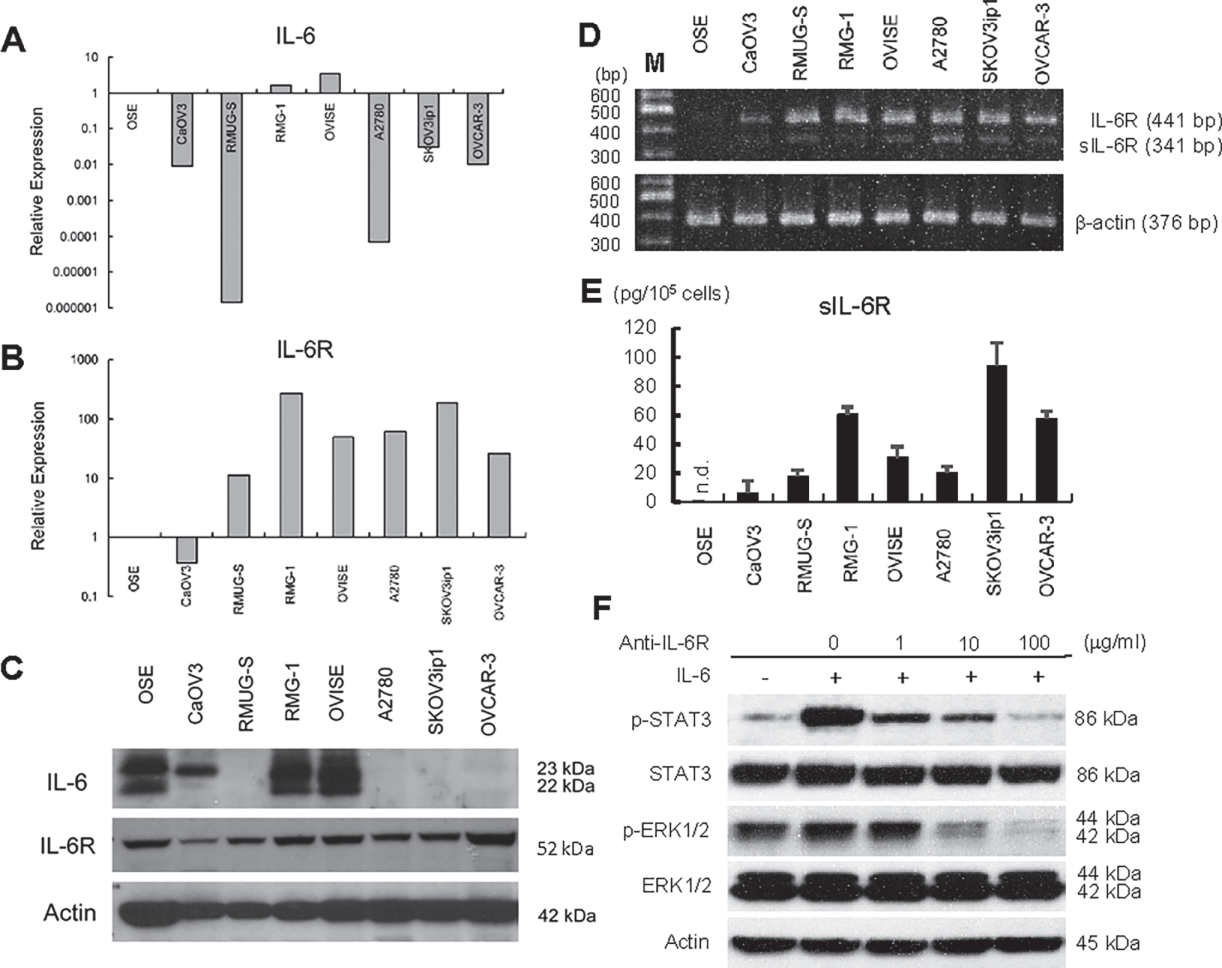
Expression of IL-6 and IL-6R in ovarian cancer cell lines. Real-time RT-PCR of IL-6 (A) and IL-6R (B). Total RNA was collected from seven different ovarian cancer cell lines using TRIzol and subjected to real-time RT-PCR. The 2^-ΔΔCT^ method was used to calculate the relative abundance with respect to GAPDH expression. Relative fold differences with respect to primary ovarian surface epithelium (OSE) are presented. (C) Western Blot. Cell lysates from seven ovarian cancer cells were resolved by SDS-PAGE and immunoblotted with an antibody against IL-6 and IL-6R. β-Actin was used as a loading control. (D) RT-PCR. RNA was collected and the expressions of full-length IL-6R (IL-6R) and soluble IL-6R (sIL-6R) expression in ovarian cancer cell lines were examined. PCR conditions were as described in Material and Methods. (E) ELISA assay of sIL-6R. Seven ovarian cancer cells (1 x 10^5^ each) were plated onto 24-well plates and cultured with 1 ml of serum-free medium for 72 h. Conditioned media were collected and the concentration of human sIL-6R was measured by ELISA. Experiments were repeated three times. n.d.; not detected. (F) Western Blot. Exogenous treatment of IL-6 activates IL-6/IL-6R signaling in ovarian cancer cell lines. SKOV3ip1 cells were stimulated with IL-6 (100 ng/mL) for 30 minutes with or without pretreatment using ranti-IL-6R antibody (1–100 μg/ml) non-immune IgG as control. Cell lysates were collected and equal amount of cell lysates (30 μg) was resolved by 10% SDS-PAGE and immunoblotted with anti–phosphorylated STAT3 (p-STAT3) antibody and anti–phosphorylated p44/42 MAPK (p-ERK1/2) antibody. The membranes were stripped and rehybridized with antibodies detecting the total forms of the protein. Blots are representative of three experiments.

Since “high” expression of IL-6R but not IL-6 affected the prognoses of patients with ovarian cancer, we quantified the expressions of IL-6 and IL-6R in ovarian cancer cell lines by real-time RT-PCR. The expression level of primary OSE cells was set as 1.0. While only two (RMG-1 and OVISE) of seven cell lines expressed high levels of IL-6 compared with OSE cells (**Fig. 2A**), six (RMUG-S, RMG-1, OVISE, A2780, SKOV3ip1 and OVCAR-3) of seven cell lines showed markedly high expression of IL-6R (11.0–270.1 fold) (**Fig. 2B**). Similar trends were confirmed by Western Blot (**Fig. 2C**). The membrane-bound form of IL6R expression is largely restricted to hepatocytes, immune cells and some tumor cells.[6] Therefore, the soluble isoform (sIL-6R) is considered to play important roles in inflammatory contexts by allowing a heightened response to IL-6 in all cell types. In order to determine whether ovarian cancer cell lines predominantly express the full-length (IL-6R) or the differentially spliced isoform that lacks the transmembrane domain (sIL-6R), we designed primers that code splicing lesions and performed RT-PCR (**Fig. 2D**). In all cell lines except OSE, we found that both IL-6R and sIL-6R were expressed. The expression of sIL-6R was further confirmed by quantitative ELISA. All ovarian cancer cell lines tested produced a certain amount of sIL-6R (6.3–94.0 pg/10^5^ cells, Fig. 2E). Thus, the treatment with IL-6 (100 ng/ml) alone drastically induced the phosphorylation of STAT3, a key downstream molecule in the IL-6/IL-6R signaling pathway, as well as that of ERK in SKOV3ip1 cells (**Fig. 2F**), and the addition of exogenous sIL-6R was not required for these phosphorylations. The pretreatment of anti-IL-6R antibody inhibited those reactions in a dose-dependent manner, indicating that IL-6R is highly expressed in certain types of ovarian cancer cells and that IL-6/IL-6R signaling exerts in a paracrine manner in these cells, even though the cells do not produce IL-6.

### Exogenous treatment of IL-6 induces proliferation, invasion and VEGF production of ovarian cancer cells

**Fig 3:**
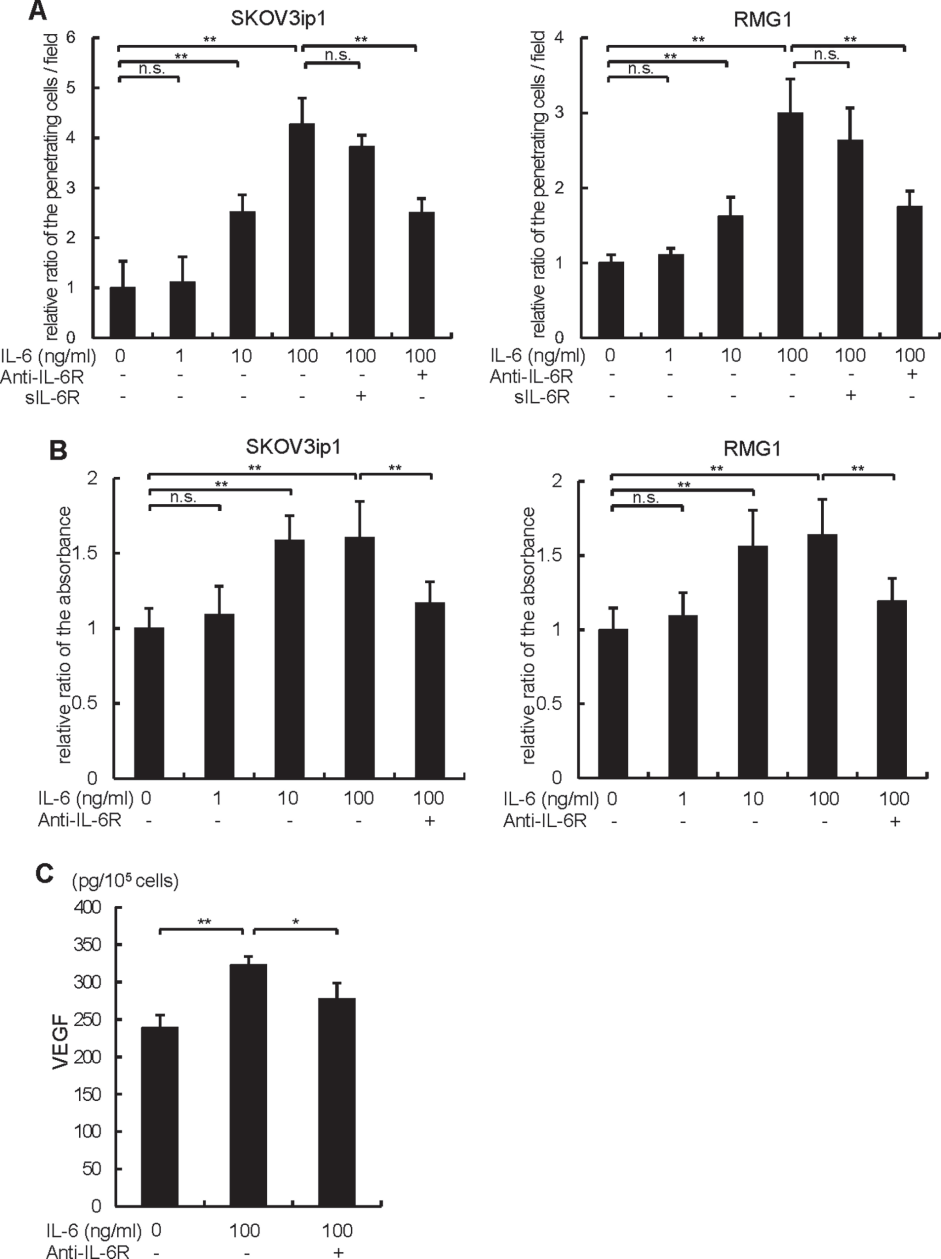
Exogenous treatment of IL-6 promotes ovarian cancer cell proliferation, invasion and VEGF production. (A) A matrigel invasion assay was done using a modified Boyden chamber system. 1 x 10^5^ of SKOV3ip1 (*left*) or RMUS-S (*right*) cells were placed on the top chamber in serum-free medium and allowed to invade for 72 h. Various concentrations of IL-6 (1–100 ng/ml) or 60 ng/ml of sIL-6R were applied in the bottom chamber as a chemoattractant. 10 μg/ml of anti-IL-6R antibody or non-immune IgG was co-treated. Non-invading cells were removed using a cotton swab, and invading cells on the underside of the filter were enumerated. Relative numbers of invading cells with respect to the control (no IL-6 treatment) are shown. (B) *In vitro* cell proliferation assay. 1 x 10^4^ cells of SKOV3ip1 (*left*) or RMG1 (*right*) cells were plated in 24-well plates in 10% FBS/DMEM for 24 h and then incubated in serum-free medium in the presence or absence of various concentrations of IL-6 (1–100 ng/ml) with or without anti-IL-6R antibody or non-immune IgG as control for 72 h. Cell proliferation was evaluated by a modified MTS assay. Cell proliferation was expressed as the ratio of the number of viable cells. (C) ELISA assay of VEGF-A. 1 x 10^5^ SKOV3ip1 cells were plated onto 6-well plates and cultured with 2 ml of serum-free medium in the presence or absence of 100 ng/ml of IL-6 for 72 h. Anti-IL-6R antibody or control IgG was co-treated. Conditioned media were collected and the concentration of human VEGF-A was measured by ELISA. Experiments were repeated three times and values are means ± SD of triplicates. n.s.; not significant, *; P < 0.05, **; P < 0.01.

Since IL-6/IL-6R signaling is known to act in a number of ways to augment cancer progression, we examined whether the exogenous treatment of IL-6 enhances proliferation, invasion and VEGF-related angiogenesis in ovarian cancer cells. For this purpose, SKOV3ip1 cells which do not express detectable protein levels of IL-6 and RMG-1 cells which express a certain level of IL-6 were employed. Both cell lines have high levels of IL-6R expression as confirmed in **Fig. 2A**. Exogenous treatment of IL-6 robustly induced cell invasion of ovarian cancer cells in a dose dependent manner (IL-6 100ng/ml; SKOV3ip1, 4.27 ± 0.53 fold, RMG-1, 2.99 ± 0.46 fold), whereas the induction was almost completely inhibited by the pretreatment with anti-IL-6R antibody (**Fig. 3A**). The addition of exogenous sIL-6R did not further promote the invasion of ovarian cancer cells induced by IL-6, suggesting that IL-6Rs (IL-6R and sIL-6R) produced from cancer cells are enough to activate IL-6/IL-6R signaling. Exogenous treatment of IL-6 also significantly enhanced the proliferation of both ovarian cancer cells in a dose dependent manner (IL-6 100ng/ml; SKOV3ip1, 1.60 ± 0.29 fold, RMG-1, 1.64 ± 0.39 fold) and the pretreatment of anti-IL-6R antibody almost abolished these enhancements (**Fig. 3B**). Next, we examined VEGF production from SKOV3ip1 cells. Exogenous treatment of IL-6 (100 ng/ml, 72h) significantly stimulated VEGF production from cancer cells (control; 239.4 ± 16.3 pg/1x 10^5^ cells, IL-6; 322.4 ± 11.8 pg/1x10^5^ cells) and the pretreatment of cancer cells with anti-IL-6R antibody significantly inhibited VEGF production (**Fig. 3C**). Collectively, exogenous treatment with IL-6 induced proliferation, invasion and VEGF production of ovarian cancer cells, even though they do not express IL-6, and co-treatment with soluble IL-6R was not required. These data suggested that in certain types of ovarian cancer cells, IL-6/IL-6R signaling can augment cancer progression in a paracrine manner.

### CD11b^+^CD14^+^ cells enhanced invasion and proliferation of ovarian cancer cells through IL-6 production

**Fig 4:**
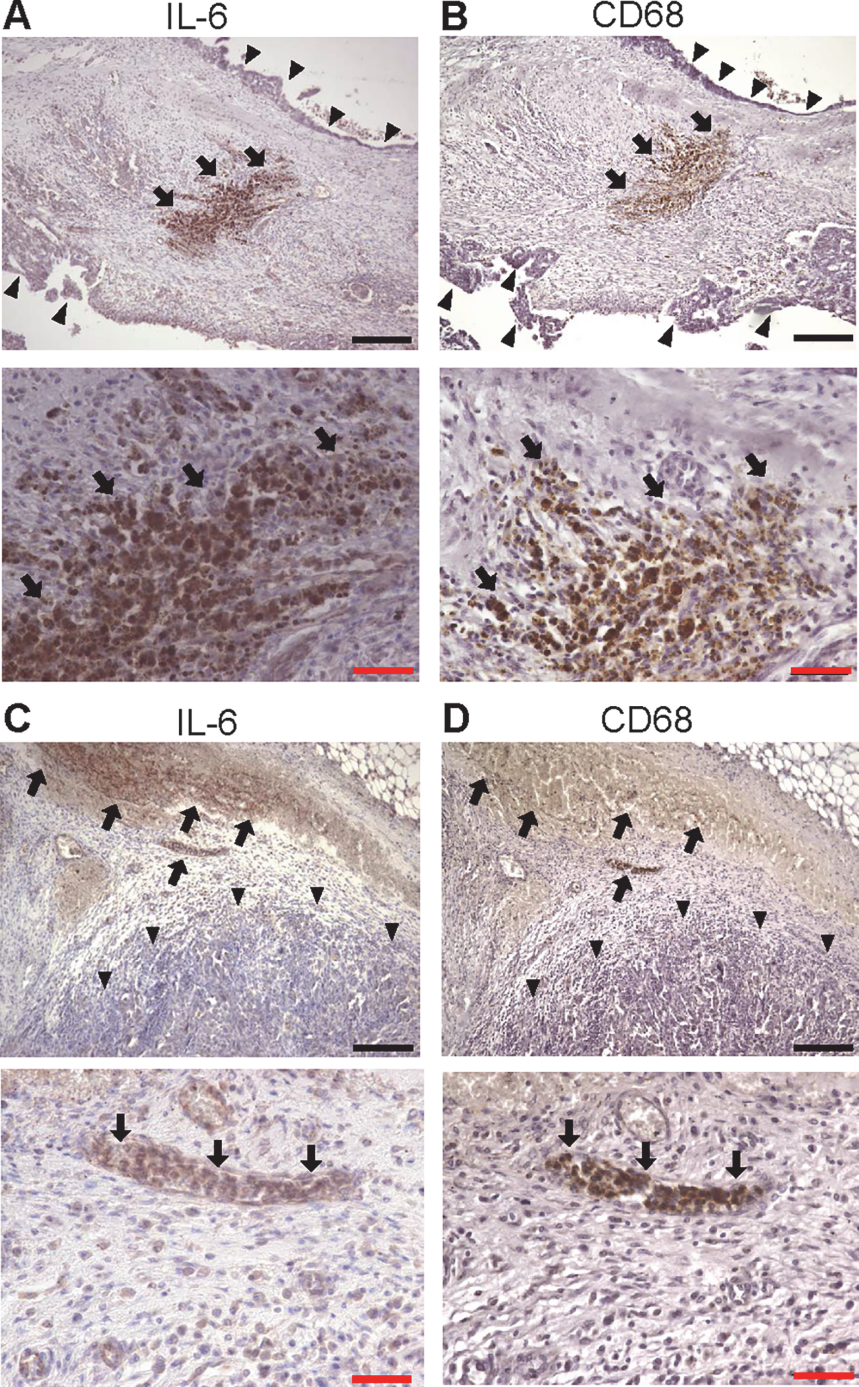
Immunohistochemical analyses of IL-6 in high-grade serous ovarian cancer tissues. Serial sections of stage III high-grade serous ovarian cancer tissues were immunostained with anti-IL-6 antibody (A, C) and anti-CD-68 antibody (B, D). IL-6 was strongly expressed in stroma, while cancer cells little expressed IL-6. (A, B) Sections from a 56 year-old female with stage IIIC high-grade serous ovarian cancer. (C, D) sections from a 63 year-old female with stage IIIC high-grade serous ovarian cancer. CD68 staining identified macrophages. Arrows indicate macrophages. Arrowheads indicate ovarian cancer cells. Original magnification, x100 (upper panels), and x400 (bottom panels). Black bar; 200 μm, red bar; 50 μm.

**Fig 5:**
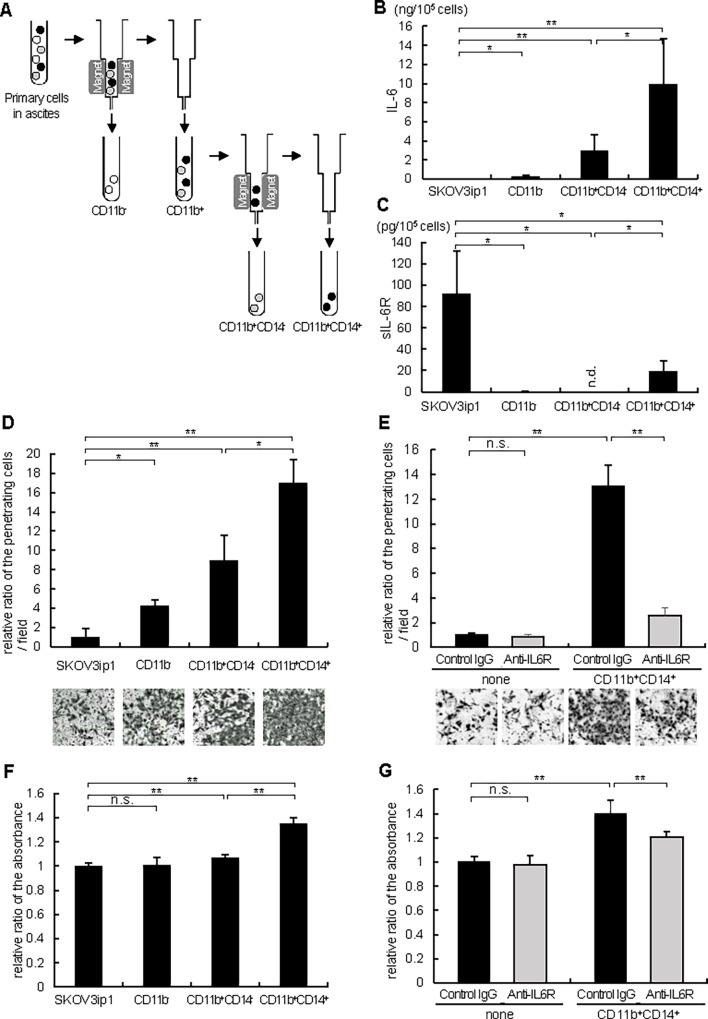
CD11b^+^CD14^+^ cells from ovarian cancer ascites promote ovarian cancer cell invasion and proliferation *via* producing IL-6. (A) The protocol of isolation of CD11b^-^, CD11b^+^CD14^-^ and CD11b^+^CD14^+^ cells using magnetic-activated cell sorting (MACS) technology (Miltenyi Biotech). ELISA assay of IL-6 (B) and sIL-6R (C). 1 x 10^5^ of SKOV3ip1 cells and primary cells indicated in the figure were plated onto 6-well plates and cultured with 2 ml of serum-free medium for 72 h. Conditioned media were collected and the concentrations of human IL-6 (B) as well as sIL-6R (C) were measured by ELISA. Experiments were repeated three times and values are means (±SD) of triplicates. (D) Matrigel invasion assay. 1 x 10^5^ SKOV3ip1 cells were placed on the upper chamber with the same number of primary cells indicated in the figure seeded on the bottom chamber as a chemoattractant, and were allowed to invade for 72 h. The relative number of invading cells when no cells were plated on the bottom chamber was set as 1.0. (E) Anti-IL-6R antibody inhibited ovarian cancer cell invasion induced by CD11b^+^CD14^+^ cells. In this experiment, the co-culture experiment in Fig. 4D was repeated with the addition of the 10 μg/ml of anti-IL-6R antibody or non-immune IgG in the bottom chamber. Representative pictures of transwells are shown in Fig. 4D and 4E (*bottom*). (F) In vitro cell proliferation assay. 1 x 10^4^ SKOV3ip1 cells were plated in 24-well plates. Thereafter, polycarbonate filters with 1-μm pores were placed onto 24-well plates and the same number of primary cells indicated in the figure were seeded as a stimulant and cells were cultured for 72 h. Cell proliferation was expressed as the ratio of the number of viable cells. (G) Anti-IL-6R antibody inhibited ovarian cancer cell proliferation induced by CD11b^+^CD14^+^ cells. In this experiment, the co-culture experiment in Fig. 4F was repeated with the addition of the 10 μg/μl of anti-IL-6R antibody or non-immune IgG in the upper chamber. Experiments were repeated three times and values are means ± SD of triplicates. n.s.; not significant, n.d.; not detected, *; P < 0.05, **; P < 0.01.

Since IL-6/IL-6R signaling contributed to ovarian cancer progression in a paracrine way, we considered that inflammatory microenvironments surrounding ovarian cancers may constitute cytokine-rich environments, especially by producing IL-6. First, we immunostained ovarian cancer tissues including surrounding stroma with anti-IL-6 antibody and found that IL-6 was strongly expressed in stroma, even if cancer cells little expressed IL-6 (**Fig. 4A** and **4C**). To identify the type of these IL-6 positive cells, we further immunostained the serial sections with anti-CD-68 (a marker of macrophage) antibody (**Fig. 4B** and **4D**). IL-6 positive cells were also CD-68 positive, suggesting that IL-6 expressing cells surrounding ovarian cancers are mainly macrophages. Next, as several previous reports demonstrated that IL-6 was elevated in the serum as well as peritoneal fluid of patients with ovarian cancer,[3, 22, 23] we focused on the primary cells present in ovarian cancer ascites. Ascites were collected aseptically from 5 different ovarian cancer patients as summarized in Table 3. Cells present in ascites were isolated by standard Ficoll-Paque density-gradient and separated into 3 groups by using magnetic-activated cell sorting; CD11b^-^ cells, CD11b^+^CD14^-^ cells and CD11b^+^CD14^+^ cells (**Fig. 5A**). CD11b^+^CD14^+^ cells represent monocytes or macrophages and CD11b^+^CD14^-^ cells represent myeloid derived cells other than macrophages (e.g. myeloid derived suppressor cells (MDSC), neutrophil). CD11b^-^cells represent primary cancer cells or other stromal cells.[24] First, the IL-6 production in each culture medium was measured by quantitative ELISA. While CD11b^-^ cells as well as SKOV3ip1 cells produced little IL-6 (CD11b^-^; 0.21 ± 0.19 ng/1x10^5^ cells, SKOV3ip1; 0.0048 ± 0.0009 ng/1x10^5^ cells), CD11b^+^CD14^+^ cells produced a high level of IL-6 compared with CD11b^+^CD14^-^ or other cells (CD11b^+^CD14^+^; 9.86 ± 4.78 ng/1x10^5^ cells, CD11b^+^CD14^-^; 2.91 ± 1.70 ng/1x10^5^ cells) (**Fig. 5B**), suggesting that IL-6 in ovarian cancer ascites is mainly produced from macrophages. The production of sIL-6R was also examined by quantitative ELISA. SKOV3ip1 and CD11b^+^CD14^+^ cells produced a high level of sIL-6R (SKOV3ip1; 91.8 pg/1x10^5^ cells, CD11b^+^CD14^+^; 19.5 pg/1x10^5^ cells), while either CD11b^-^ cells or CD11b^+^CD14^-^ cells little produced sIL-6R (**Fig. 5C**). Next, *in vitro* Matrigel-coated transwell assays were performed by co-culturing equal numbers of primary cells to the lower chamber as a chemoattractant. The co-culture of CD11b^+^CD14^+^ cells drastically induced *in vitro* invasion of SKOV3ip1 cells compared with control or other cells (16.9 ± 2.5 fold compared with control (when no cells were co-cultured)) (**Fig. 5D**). This enhanced invasion of cancer cells was almost inhibited by the treatment with anti-IL-6R antibody, while the antibody did not affect the cell invasion when no cells were co-cultured (**Fig. 5E**). Cell proliferation was assayed by a modified MTS assay. The co-culture of CD11b^+^CD14^+^ cells significantly induced cell proliferation of SKOV3ip1 cells compared with the control (1.35 ± 0.05 fold compared with control (when no cells were co-cultured)) (**Fig. 5F**). This proliferation was significantly inhibited by the treatment with anti-IL-6R antibody, while anti-IL-6R antibody alone did not affect the proliferation of SKOV3ip1 cells (**Fig. 5G**). Collectively, among the primary cells in ovarian cancer ascites, CD11b^+^CD14^+^ cells, which represent macrophages, induced cell invasion and the proliferation of ovarian cancer cells, at least partly, through the production of IL-6.

**Table 3:** Characteristics of the patients whose ascites were collected and used for analyses.

Patient No.	Age	Histrogic subtype	FIGO stage
1	20	Immature teratoma	IIc
2	46	Clear cell carcinoma	Ic
3	55	Carcinosarcoma	IV
4	61	Undifferentiated adenocarcinoma	IIIc
5	67	Endometrioid adenocarcinoma	IIIa

### CD11b^+^CD14^+^ cells were predominantly M2-polarized macrophages

**Fig 6:**
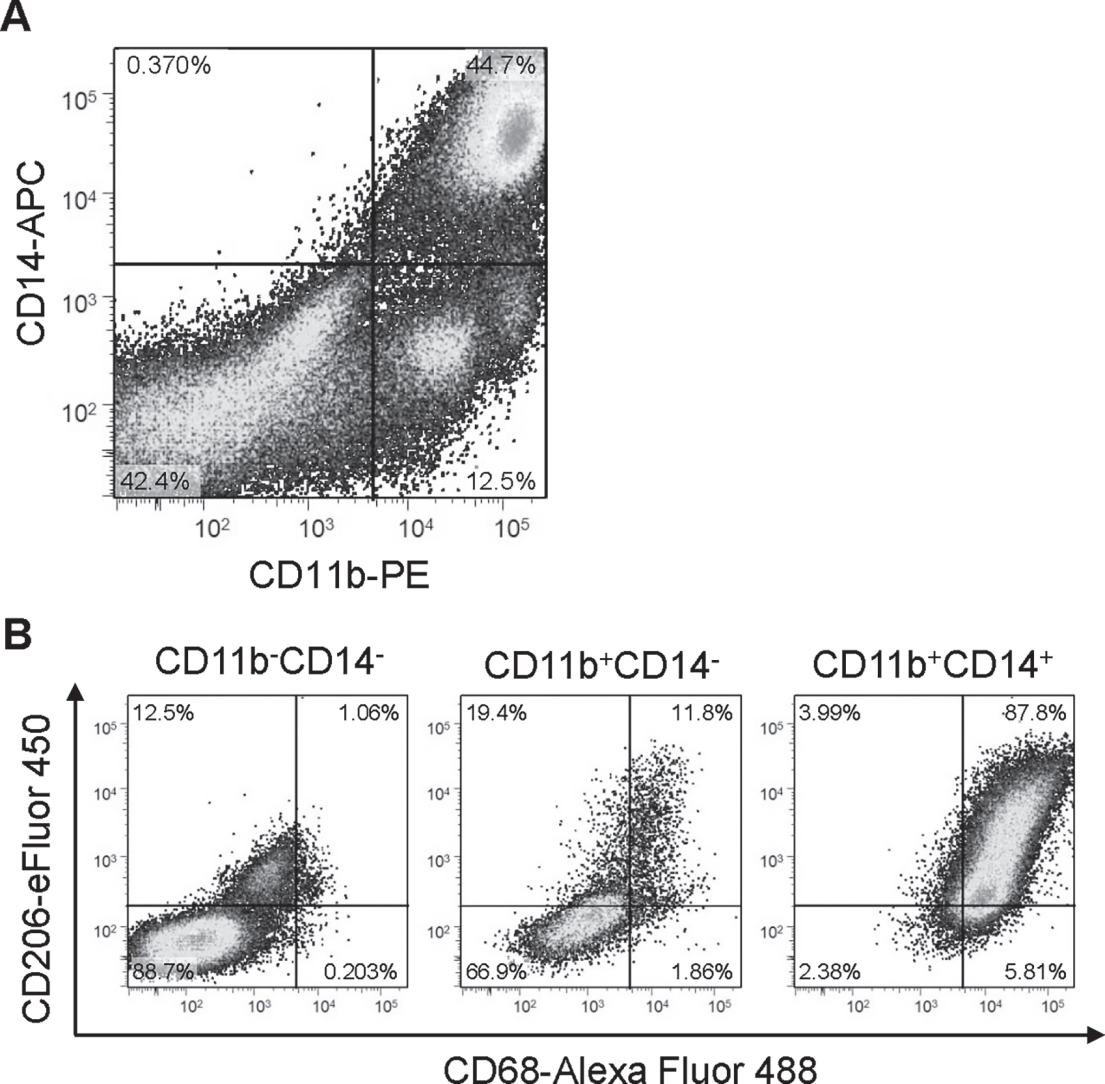
Characterization of primary cells from ovarian cancer ascites. (A) FACS analyses. Ovarian cancer ascites were collected aseptically, and cells were isolated by standard Ficoll-Paque density-gradient. Thereafter, cells were labelled with cell surface markers CD11b (PE, x-axis) and CD14 (APC, y-axis). Cells were divided into 3 groups, CD11b^-^CD14^-^, CD11b^+^CD14^-^ and CD11b^+^CD14^+^ cells. (B) The majority of CD11b^+^CD14^+^ cells are M2-polalized macrophages. These three populations of cells were further labeled with cell surface markers CD68 (Alexa Fluor-488, x-axis) and CD206 (eFluor-450, y-axis). 87.8% of CD11b^+^CD14^+^ cells (*right*) were CD68 and CD206 positive.

In order to further elucidate the properties of primary cells in ovarian cancer ascites, cells were sorted by FACS with anti-CD11b and CD14 antibodies (**Fig. 6A**). Cells were divided into 3 groups; CD11b^-^CD14^-^, CD11b^+^CD14^-^, CD11b^+^CD14^+^ cells. CD11b^+^CD14^+^ cells were further analyzed for the expression of surface markers linked to macrophage activation or polarization using anti-CD68 and CD206 antibodies. The majority (87.8%) of CD11b^+^CD14^+^ cells were CD-68, a surface marker of macrophages, and CD-206, a surface marker of positive M2-polarized macrophages, suggesting that IL-6 producing cells in ovarian cancer ascites are predominantly M2-polarized macrophages, TAMs (**Fig. 6B**).

## Discussion

**Fig 7:**
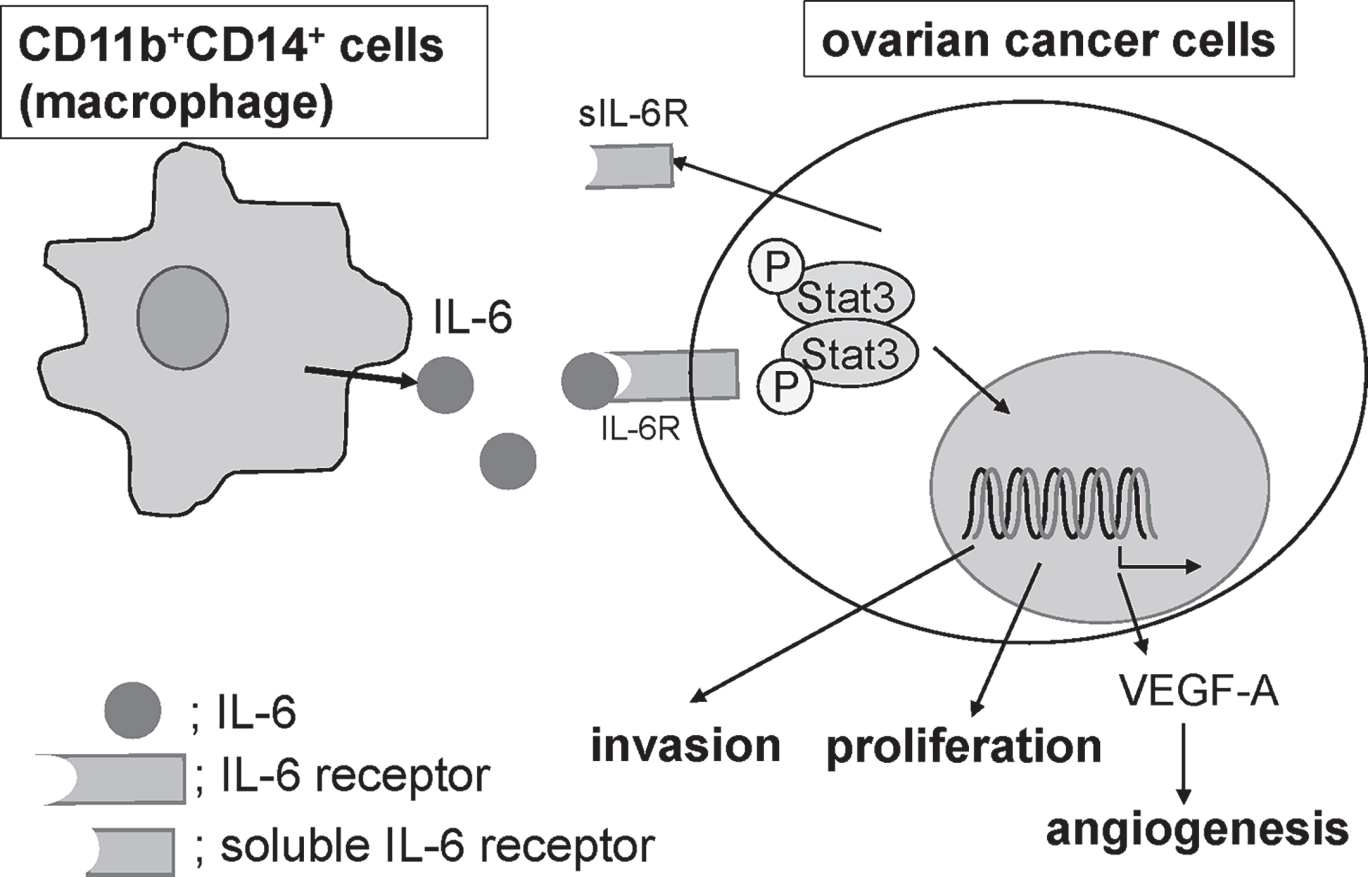
Proposed paracrine mechanism. CD11b^+^CD14^+^ cells, which are predominantly macrophages, are one of the major sources of IL-6 production in an ovarian tumor microenvironment and promoted ovarian cancer invasion, proliferation and angiogenesis.

The treatment of ovarian cancer patients continues to be challenging. In this study, we first revealed that a high-level of IL-6R expression in cancer tissues is an independent prognosis factor of ovarian cancer patients. *In vitro* analyses using seven ovarian cell lines revealed that six of seven cell lines overexpressed IL-6R compared with a primary OSE, indicating that IL-6/IL-6R signaling exerts in a paracrine manner in certain types of ovarian cancer cells, as confirmed by the data that exogenous treatment of IL-6 induced proliferation, invasion and VEGF production of ovarian cancer cells which do not express detectable levels of IL-6. From ovarian cancer ascites, CD11b^-^, CD11b^+^CD14^-^ and CD11b^+^CD14^+^ cells were isolated and we found that CD11b^+^CD14^+^ cells expressed significantly high levels of IL-6 compared with other populations of cells and FACS analyses revealed that approximately 90% of CD11b^+^CD14^+^ were mannose receptor, CD206 positive, indicating that these populations of cells are predominantly M2-polarized macrophages, TAMs. When CD11b^+^CD14^+^ cells were co-cultured with ovarian cancer cells, the invasion as well as the proliferation of cancer cells were robustly promoted and these promotions were almost completely inhibited by the pretreatment with anti-IL-6R antibody, while anti-IL-6R antibody did not affect ovarian cancer cell invasion and proliferation without CD11b^+^CD14^+^ cells, indicating that TAMs enhance ovarian cancer proliferation or invasion by secreting a certain amount of IL-6. Proposed paracrine mechanism is shown as **Fig. 7**.

There is increasing evidence that IL-6/IL-6R signaling may play a significant role in the progression of various cancers, including ovarian carcinomas. Several observations have documented that IL-6 levels in the serum and peritoneal fluid from patients with ovarian cancer are increased and that high levels of IL-6 are significantly associated with poor prognosis and survival of the patients.[3, 4, 22, 25] However, there remains a debate as to the source of the IL-6 in ovarian cancer patients. In 2011, Coward et al. examined IL-6 and IL-6R expression in tissue microarrays from 221 ovarian cancer biopsies and showed that high IL-6 expression was significantly associated with shorter progression free survival.[26]　More recently, Reinartz et al. analyzed the polarization of macrophages in ovarian cancer ascites and showed that surface expression of the M2 marker CD163 on macrophages was inversely associated with progression free survival of the patients and that elevated CD163 expression was associated with increased IL-6 levels, which were consistent with our findings.[27] It appears reasonable that IL-6 is secreted either directly from ovarian cancer cells or from stromal cells present in the ovarian cancer microenvironments. Further elucidation will be needed to clarify the source and role of IL-6 in ovarian cancer microenvironments. Our study showed that enriched IL-6 secretion from M2-polarized macrophages in ascites promotes ovarian cancer progression through “high” expression of IL-6R in ovarian cancer cells. As to IL-6R expression, although some have reported that increased IL-6R expression has been observed in ovarian cancer cells,[6, 28] no study has shown the prognostic impact of IL-6R expression in ovarian cancer tissues on patients. To our knowledge, our report is the first to indicate that “high” IL-6R expression is an independent prognostic factor for ovarian cancer patients.

The increasing evidence regarding the molecular biology of IL-6 and its interrelations with human cancer cells as well as their microenvironments have led to the development of novel antibody-based therapies. Several IL-6 antibodies have been developed in recent years and evaluated in clinical trials, such as the anti-IL-6 chimeric antibody, CNTO 328 (siltuximab) developed by Centocor (Horsham, PA).[7] Siltuximab has been used in clinical trials and found to be well tolerated in patients having different cancers, including ovarian cancer. A phase 2 single-arm clinical trial of siltuximab against women with advanced platinum-resistant ovarian cancer was reported.[26] Twenty patients were recruited (19 had high-grade serous ovarian cancer and one had clear cell ovarian cancer). Of the 18 evaluable patients, one patient was alive 127 weeks after trial entry, although 16 died and one was lost to follow-up. However, in eight patients, stable disease was achieved, lasting 6 months or more in 4 patients. Stone RL, et al. showed that anti–IL-6 antibody treatment significantly reduced platelet counts in tumor-bearing mice and enhanced the therapeutic efficacy of paclitaxel in mouse models of epithelial ovarian cancer.[25] More recently, a novel high-affinity fully human anti-IL-6 mAb, 1339 was developed[29] and has shown promise in preclinical models of several cancers.[7] As for targeting IL-6R, tocilizumab has been reported to be effective in treating oral squamous cell carcinoma [9] and renal cell carcinoma [30] in preclinical animal models. In a case report, the treatment with tocilizumab to primary lung cancer in a 75-year-old man greatly and rapidly improved cachexia-related symptoms and provided 9 months of survival.[31] Although tocilizumab has not been previously reported as beneficial for patients with cancer, we believe that targeting IL-6R with tocilizumab would be worth evaluating as a novel therapeutic agent for ovarian cancer, since we showed here that “high” IL-6R expression is an independent prognostic factor and antagonizing IL-6R significantly inhibited ovarian cancer progression and tumor-related angiogenesis regardless of IL-6 expression in cancer cells.

New active molecules have been developed and several studies have been conducted or are underway against ovarian cancer, of which the most successful to date is the anti-VEGF antibody, bevacizumab.[32] Although bevacizumab has shown significant efficacies not only as a first-line therapy combined with paclitaxel plus carboplatin but also as a treatment for relapsed cases with ovarian cancer, bevacizumab has failed to show clinical benefits on overall survival of patients.[32] Considering the limited efficacies of molecular-targeted therapies reported so far, it appears obvious that the mere inhibition of IL-6/IL-6R signaling would be insufficient for a pronounced response unless combined with apoptosis-inducing drugs for ovarian cancer treatment and IL-6/IL-6R inhibitors should be used as adjuvants along with cytotoxic chemotherapies in the clinical setting. For these reasons, we assume that tocilizumab would be an ideal candidate because it has proven sufficiently safe for patients and can be combined with current chemotherapies.[11] Currently, tocilizumab is clinically used to treat Castleman’s disease and rheumatoid arthritis at a dose of 8 mg/kg administered by intravenous drip infusion at 2-week intervals. Further study is required to identify appropriate drug administration routes or doses before clinical application.

In this study, we showed that IL-6 in ovarian cancer ascites is mainly derived from CD11b^+^CD14^+^CD206^+^ cells, M2-polalized macrophages, TAMs. Previously, Duluc D, et al. reported that ovarian cancer ascites contained high concentrations of leukemia inhibitory factor (LIF) and IL-6 and that both skew monocyte differentiation into TAM like cells, suggesting an important role of IL-6 in TAM generation.[10] More recently, Dijkgraaf EM et al. reported that treatment with cisplatin or carboplatin induced the production of IL-6 from ovarian cancer cell lines and increased the potency of cell lines to skew monocytes into M2 macrophage differentiation. This M2 differentiation was prevented by treatment with tocilizumab, suggesting that this antibody might increase the clinical effect of platinum-based chemotherapy.[33] However, in our study, all samples of primary cells from ovarian cancer ascites were collected before chemotherapies and it appears that the enriched IL-6 accumulation in ascites is seen at advanced stages regardless of chemotherapies. At least, it seems that IL-6 accumulation in ascites induces skewing of monocytes into TAMs and differentiated TAMs further produce IL-6, which leads to the invasion, proliferation and angiogenesis of ovarian cancer cells.

In conclusion, we showed that “high” IL-6R is an independent prognostic factor of ovarian cancer patients and IL-6 from TAMs present in ascites promotes ovarian cancer invasion, proliferation and angiogenesis. The ablation of IL-6R function by tocilizumab led to a substantial decrease in tumor progression, suggesting the potential of tocilizumab as a therapeutic strategy for ovarian cancer treatment. In light of various publications highlighting the importance of IL-6/IL-6R signaling in ovarian tumor biology and the results described here, we are supportive of clinical trials to study whether antagonizing IL-6R is a viable treatment strategy for ovarian cancer.
